# *VacA* and *cagA* genotypes of *Helicobacter pylori* isolated from raw meat in Isfahan province, Iran

**Published:** 2017-03-15

**Authors:** Ali Gilani, Vadood Razavilar, Nordahr Rokni, Ebrahim Rahimi

**Affiliations:** 1 *PhD Candidate, Department of Food Hygiene and Quality Control, Science and Research Branch, Islamic Azad University, Tehran, Iran; *; 2 *Department of Food Hygiene and Quality Control, Science and Research Branch, Islamic Azad University, Tehran, Iran;*; 3 *Department of Food Hygiene and Quality Control, College of Veterinary Medicine, Shahrekord Branch, Islamic Azad University, Shahrekord, Iran.*

**Keywords:** Butchery, Genotype, *Helicobacter pylori*, Meat, Slaughterhouse

## Abstract

Foods with animal origins play a substantial role in the transmission of *Helicobacter pylori*. The present investigation was carried out to study the *vacA* and *cagA* genotypes status of *H. pylori* isolated from various types of meat samples. Two hundred and twenty meat samples were collected and cultured. *H. pylori*-positive strains were analyzed for the presence of *vacA* and *cagA* genotypes. Eleven out of 220 (5.00%) samples were positive for *H. pylori*. Findings were confirmed by nested PCR. Prevalence of *H. pylori* in the meat samples of slaughterhouses and butcheries were 72.20% and 27.70%, respectively. The most commonly detected genotypes in the meat samples of slaughterhouses and butcheries were *vacA* *m1a* (66.66%) and *vacA s1a* (37.50%), respectively. The *S1am1a* was the most commonly detected genotype. Meat sampled from butcheries had the higher prevalence of *H. pylori* and its genotypes than those of slaughterhouses (*p* < 0.05). Results showed that meat samples could be the potential sources of virulent strains of *H. pylori*. Application of sanitary measures in the storage, transportation and sale of meat is essential for reducing the levels of *H. pylori* cross contamination.

## Introduction

Meat as the most valuable livestock product is composed of protein and amino acids, minerals, fats and fatty acids, vitamins and other bioactive components which are beneficial for human health. From the nutritional point of view, meat’s importance is derived from its high quality protein, containing all essential amino acids and highly bioavailable minerals and vitamins. In addition, presence of essential fatty acids multiplies the nutritional value of this food.^1^^,^^2^ In keeping with this, meat can convert into the luscious, energetic and graceful products like sausage and hamburger. Due to the high consumption rate of meat and meat products in the world, observation of sanitary conditions is critical in their production and distribution. 

*Helicobacter pylori* is a microaerophilic gram negative and spiral shaped bacterium which its main reservoir is humans, predominantly the human stomach. It colonizes most of the population, making it one of the most provocative bacteria in the world. It is the most suspected cause of peptic ulcer disease, duodenal ulcer, type B gastritis, gastric adenocarcinoma and mucosa-associated lymphoid tissue and lymphoma.^3^ It has been estimated that 17 to 86.00% of hospitalized patients with peptic ulcers were infected with *H. pylori*.^3^^-^^5^

The main routes of *H. pylori* infection have not been identified yet^2^. However, it is prospective that *H. pylori* infection occurs throughout childhood or adolescence both in developed and developing countries.^5^ The main routes for transmission of *H. pylori* are person to person contacts especially fecal-oral or oral-oral.^6^

The role of foods in the transmission of *H. pylori* is still unknown but there were several novel investigations focused on the identification of this bacterium in various types of food samples.^6^^-^^10^ Suitable conditions like appropriate pH, moisture and temperature cause *H. pylori* to be easily survive in various types of foods including vegetable, salad, milk and meat.^8^^,^^10^

To appraise the pathogenicity of *H. pylori*, evaluation of latent virulence factors and genotypes is essential. The most commonly important virulence factors among *H. pylori* strains of different clinical outcomes of human and animal are the vacuolating cytotoxin (*vacA*) and cytotoxin associated gene (*cag*).^8^^,^^11^^,^^12^ These genes usually induce adhesion and invasion to the gastric epithelial cells.^13^^,^^14^ The *vacA* belongs to the group of genes with variable genotypes or structures. This gene is associated with injury to epithelial cells. The *vacA* gene is polymorphic, comprising variable signal regions (type *s1* or *s2*) and mid-regions (type *m1* or *m2*). The *s1* type is further subtyped into *s1a*, *s1b *and *s1c *subtypes and the *m1* into *m1a* and *m1b *subtypes. The mosaic combination of *s* and m-region allelic types determines the particular cytotoxin and consequently, the pathogenicity of the bacterium.^13^^-^^15^

The* Cag* pathogenicity island (PAI) has been shown to be involved in persuading inflammation, ulceration and carcinogenesis.^14^ The *cagA* gene has been detected in the specimens taken from the severe cases of peptic ulcer.^10^^-^^14^ Genotyping using these virulence markers is considered as one of the best approaches for study of correlations between *H*. *pylori *isolates from different samples.

Data on the epidemiology and transmission of *H. pylori* are extremely significant to prevent its distribution and identify high-risk populations, especially in areas that have high rates of gastritis, peptic ulcers and gastric cancer like Iran.^8^^-^^12^^,^^15^ Considering the unclear epidemiological aspects of *H. pylori* in meat and meat products and due to the high prevalence of *H*. *pylori *all around the world, the present investigation was carried out in order to study the exact status of *vacA* and *cagA* genotypes of *H. pylori* isolated from various types of meat and meat products. 

## Materials and Methods

**Sample collection. **From July to September 2015, overall 220 meat samples including beef (n = 80), mutton (n = 70) and chevon (n = 70) were collected from slaughter-houses and butcheries of various parts of Isfahan province, Iran. Samples (100 mg, in sterile glass containers) were transferred to laboratory at 4 ˚C and then refrigerated in plastic bags; information about dates of production and of assigned shelf lives was not presented. 

**Isolation of** ***H. pylori*** **. **Each homogenized sample (25 g) was added to 225 mL of Wilkins Chalgren anaerobe broth (Oxoid Ltd., Basingstoke, UK) supplemented with colistin methanesulfonate (30 mg L^-1^) and 5% of horse serum (Sigma, St. Louis, USA) and 30 mg L^-1 ^nalidixic acid, 10 mg L^-1 ^vancomycin, 100 mg L^-1 ^cycloheximide and 30 mg L^-1 ^trimethoprim (Sigma) and incubated for 7 days at 37 ˚C with shaking under microaerophilic conditions (5% oxygen, 85% nitrogen and 10% CO_2_) using MART system (Anoxamat, Lichtenvoorde, The Netherland). Then, 0.10 mL of the enrichment selective broth was plated onto Wilkins Chalgren anaerobe agar (Oxoid) supplemented with 5% of defibrinated horse blood and 30 mg L^-1^ colistin methane-sulfonate, 100 mg L^-1^ cycloheximide 30 mg L^-1^ nalidixic acid, 30 mg L^-1^ trimethoprim, and 10 mg L^-1^ vancomycin (Sigma) and incubated for seven days at 37 ˚C under micro-aerophilic conditions (5% oxygen, 85% nitrogen, and 10% CO_2_) using MART system (Anoxamat). For comparison, a reference strain of *H. pylori* (ATCC 43504) was employed.

**DNA extraction and nested PCR amplification. **Typical colonies of *H. pylori* were further identified using nested-PCR method. Genomic DNA was extracted from typical colonies using a DNA extraction kit for cells and tissues (Fermentas, St. Leon-Rot, Germany) according to the manufacturer’s instructions and its density was assessed by optic densitometry. The first and second steps of PCR was performed based on the method described previously.^16^

**Genotyping of** ***vacA*** **and** ***cagA*** **genes of** ***H. pylori*** **. **Presence of the *vacA *and *cagA* alleles were determined using PCR technique.^17^^,^^18^ All PCR reactions were done using the programmable thermal cycler (Eppendorf Co., Hamburg, Germany). All runs included one negative DNA control consisting of PCR grade water and two or more positive controls (26695, J99, SS1, Tx30, 88-23 and 84-183). A PCR method used for amplification of various genotypes of *vacA* was done with a total volume of 50 µL including 2 mM Mgcl_2_, 1 µM of forward primer, 1 µM of reverse primer, 5 µL PCR buffer 10X, 200 µM dNTP (Fermentas), 1 U Taq DNA polymerase (Fermentas) and 2.50 µL DNA template. The DNA was then amplified by 32 successive cycles of denaturation at 95 ˚C for 45 sec, primer annealing at 64 ˚C for 50 sec and DNA chain extension at 72 ˚C for 70 sec. Similar PCR reaction was done for the *cagA* genotype. Amplified DNA of *cagA* PCR method was then amplified by 32 successive cycles of denaturation at 95 ˚C for 60 sec, primer annealing at 56 ˚C for 60 sec and DNA chain extension at 72 ˚C for 60 sec.

**Gel electrophoresis. **The PCR amplification products (10 μL) were subjected to electrophoresis in a 2% agarose gel in 1X TBE buffer at 80 V for 30 min, stained with SYBR Green and images were obtained in a UVI doc gel documentation systems (Uvitec Limited, Cambridge, UK). The PCR products were identified by 100 bp DNA size marker (Fermentas). 

**Statistical analysis. **Data were transferred to Microsoft Excel spreadsheet (version 15; Microsoft Corp., Redmond, USA) for analysis. Using statistical software (version 16; SPSS Inc., Chicago, USA), Chi-square test and Fisher’s exact two-tailed test analysis were performed and differences were considered significant at values of *p* < 0.05. Distribution of *H. pylori* genotypes isolated from meat samples was statistically analyzed.

## Results

Table 1 represents the total prevalence of *H. pylori* in various types of meat samples. Eleven out of 220 (5.00%) meat samples were positive for the *H. pylori* in the culture-based method. The results of culture method were confirmed by nested PCR technique. Total prevalence of *H. pylori *in meat of slaughterhouses and butcheries were 2.72% and 7.27%, respectively. 

**Table 1:** Total prevalence of Helicobacter pylori in meat samples in Isfahan province, Iran

**Type of samples**		**No. of samples **	**No. of ** ***H. pylori*** ** in culture (%)**	**No. of ** ***H. pylori*** ** confirmed in nested PCR (%)**
**Slaughterhouses**	**Beef**	40	-	-
	**Mutton**	35	2 (5.71)	2 (5.71)
	**Chevon**	35	1 (2.85)	1 (2.85)
	**Total**	**110**	**3 (2.72)**	**3 (2.72)**
**Butcheries**	**Beef**	40	2 (5/00)	2 (5/00)
	**Mutton**	35	4 (11.42)	4 (11.42)
	**Chevon**	35	2 (5.71)	2 (5.71)
	**Total**	**110**	**8 (7.27)**	**8 (7.27)**

Totally, mutton from butcheries (11.42%), mutton from slaughterhouses (5.71%) and chevon from butcheries (5.71%) had the highest prevalence of *H. pylori*. The meat samples taken from butcheries had the higher prevalence of *H. pylori* than those of slaughterhouses (*p* < 0.05). 

Table 2 shows the distribution of various genotypes in *H. pylori* strains of meat and meat products. The most commonly detected genotypes in the meat samples taken from butcheries and those of slaughterhouses were *vacA m1a* (66.66%) and *vacA s1a* (37.50%), respectively. The *H. pylori* strains of butcher’s meat samples harbored the higher prevalence of all studied genotypes than those of slaughterhouses (*p* < 0.05). 

**Table 2:** Distribution of various genotypes in *Helicobacter pylori* strains of meat samples

**Types of samples ** **(No. positive samples)**		**Distribution of genotypes (%)**							
		***VacA***							***CagA***
		***S1a***	***S1b***	***S1c***	***S2***	***M1a***	***M1b***	***M2***	
**Slaughterhouses**	**Mutton (2)**	1 (50.00)	-	-	-	1 (50.00)	-	-	-
	**Chevon (1)**	-	-	-	-	1 (100)	-	-	-
	**Total (3)**	**1 (33.33)**	**-**	**-**	**-**	**2 (66.66)**	**-**	**-**	**-**
**Butcheries**	**Beef (2)**	1 (50.00)	-	-	-	1 (50.00)	-	-	-
	**Mutton (4)**	1 (25.00)	-	-	1 (25.00)	-	-	1 (25.00)	1 (25.00)
	**Chevon (2)**	1 (50.00)	-	-	-	-	-	-	1 (50.00)
	**Total (8)**	**3 (37.50)**	**-**	**-**	**1 (12.50)**	**1 (12.50)**	**-**	**1 (12.50)**	**2 (25)**

Table 3 reveals the distribution of combined genotypes in the *H. pylori* strains of meat and meat products. The *S1am1a* was the most commonly detected genotype in the *H. pylori* strains of all studied samples. The most commonly detected genotype in the *H. pylori* strains of slaughter-houses and butcheries were *s1am1a*, too. 

**Table 3:** Distribution of combined genotypes in the *Helicobacter pylori* strains of meat samples

**Types of samples ** **(No. positive samples)**		**Distribution of combined genotypes of ** ***VacA*** ** (%)**										
		***s1am1a***	***s1am1b***	***s1am2***	***s1bm1a***	***s1bm1b***	***s1bm2***	***s1cm1a***	***s1cm1b***	***s1cm2***	***s2m1a***	***s2m1b***
**Slaughterhouses**	**Mutton (2)**	1 (50.00)	-	-	-	-	-	-	-	-	-	-
	**Chevon (1)**	-	-	-	-	-	-	-	-	-	-	-
	**Total (3)**	**1(33.33)**	**-**	**-**	**-**	**-**	**-**	**-**	**-**	**-**	**-**	**-**
**Butcheries**	**Beef (2)**	1 (5000)	-	-	-	-	-	-	-	-	-	-
	**Mutton (4)**	-	-	1 (25.00)	-	-	-	-	-	-	-	-
	**Chevon (2)**	-	-	-	-	-	-	-	-	-	-	-
	**Total (8)**	**1(12.50)**	**-**	**1(12.50)**	**-**	**-**	**-**	**-**	**-**	**-**	**-**	**-**

## Discussion

The results of the present investigation revealed that the meat samples of slaughterhouses and butcheries were contaminated with virulent strains of *H. pylori*. We found that the meat samples of butcheries had the higher prevalence of *H. pylori*. However, the main reason for this finding is uncertain but it seems that cross contaminations of meat samples after slaughter are maybe the main factor for the higher prevalence of *H. pylori* in butcheries. Increase in the prevalence of human-based genotypes in the meat samples from butcheries is another reason for the possibility of cross contamination. In fact, meat washing, storing and transporting are the main stages which may increase the prevalence of *H. pylori* contamination. Reports showed that *H. pylori* can survive in water.^19^^,^^20^ In addition, the number of investigations which were focused on the isolation of *H. pylori* from the water samples are significant.^19^^,^^20^ The lack of drinking water for washing of animal carcass and using unsanitary water in slaughterhouses is one of the main factors for presence of *H. pylori* in meat samples. Transmission of *H. pylori* strains form the hands of infected staff and also butcheries is another reason for high prevalence of *H. pylori*. Possibility for contamination of animal carcasses in various stages of slaughter with intestinal contents, blood, feces, wool of animal and also slaughterhouse equipment such as knives, saws and hands of the meat inspector’s and sometimes butcheries attended for meat purchasing can be considered among other causes of meat contamination. The presence of exotic animals such as cats and birds in the line of slaughter is another factor which should not be ignored. 

To our best knowledge, this is the first prevalence report of isolation and genotyping of *H. pylori* in various types of raw meat samples in the world. Our results revealed that 2.72% of meat samples of slaughterhouses and 7.27% of meat samples of butcheries were contaminated with *H. pylori* strains. In comparison with other researches which were conducted on food staffs, the prevalence of *H. pylori* in our study was lower than dairy products (19.20%),^8^ vegetable and salad (10.86%),^9^ vegetables (13.68%),^10^ restaurant salad (14.00%)^10^ and milk (12.50%).^21^ As far as we know, the prevalence found in this study is the lowest report for *H. pylori* in various types of food stuffs in the world. 

Several studies have been focused on the detection of *H. pylori* in foods with animal origins.^23^^-^^26^ An Italian investigation^24^ showed that more than 25.00% of foods with animal origin were contaminated with *H. pylori*. In a study conducted in Japan, 72.20% of various types of animal-based foods were positive for *H. pylori*.^22^ Despite other foods with animal origins,^22^^-^^25^ prevalence of *H. pylori* in cattle, sheep and goat meat samples of our study was considerable. Prevalence of bacterium in Greek^24^ and USA^25^ was 20.00% and 60.00%, respectively, which was higher than our results in Iran. In a study by Mhaskar *et al*., the risk factors of *H. pylori *infection and peptic ulcer diseases were studied.^26^ They showed that meat consumption (OR: 2.35, 95% CI: 1.30 to 4.23), eating restaurant food (OR: 3.77, 95% CI: 1.39 to 10.23) and drinking nonfiltered or non-boiled water (OR: 1.05, 95% CI: 1.01 to 1.23) were the main risk factors for *H. pylori* infection which can indirectly support the results of our investigation about considerable prevalence of *H. pylori* in meat. Webberley *et al.* reported that meat-eater had the highest levels of anti-*H. Pylori* IgG than vegans which can show the impact of meat-eating as a risk factor for occurrence of *H. pylori* infection.^6^

The second part of our investigation was focused on the *vacA *and *cagA *genotype status of *H. pylori *isolates of meat samples. Our results showed that *cagA*, *vacA s1a*, *vacA m1a*, *vacA s2 *and *vacA m2* were the most commonly detected genotypes. In addition, *s1am1a*, *s1am2*, *s2m1a* and *s2m2* were the most commonly detected combined genotypes. Unfortunately, the number of studies which were focused on the genotyping of *H. pylori* in food stuffs is low. A recent Iranian investigation conducted by Yahaghi *et al.* showed that from 380 vegetable and 50 salad samples, 52 (13.68%) and 7 (14.00%) were positive for *H. pylori*, respectively.^10^ The most commonly detected genotypes in their investigation were *oipA* (86.44%), *cagA* (57.62), *vacA* *s1a* (37.28%) and *vacA m1a* (30.50%). Their results showed that *vacA s1c* had the lowest prevalence which was similar to our findings. Mousavi *et al.* showed that the most commonly detected genes in the *H. pylori* strains recovered from milk and dairy products were *cagA* (76.60%) and *vacA* (75.00%) which was similar to our findings.^8^ Hemmatinezhad *et al.* reported that 13.45% of ready to eat food samples were positive for *H. pylori*. Meat-based ready to eat food samples were the most contaminated.^27^ They revealed that the most commonly detected genotypes were *vacA* *s1a* (78.37%), *vacA* *m2* (75.67%), *vacA* *m1a* (51.35%) and *cagA* (41.89%) which was similar to our findings. They showed that *s1am2* (70.27%), *s1am1a* (39.18%) and *m1am2* (31.08%) were the most commonly detected combined genotypes which was similar to our investigation. In a study which was conducted by Saeidi and Sheikhshahrokh 820 meat and milk samples were studied for presence of *H. pylori* and *vacA* genotypes.^28^ They showed that the prevalence of *H. pylori* in meat samples was 26.25% which was higher than our results. Total prevalence of *H. pylori* in cow, sheep, goat, buffalo and camel meat samples was 25.00%, 37.00%, 22.00%, 28.00% and 14.00%, respectively. *S1a* and *m1a* were the most commonly detected genotypes. The most commonly detected combined genotypes were *m1as1a* (68.52%), *m1as1b* (60.40%), *m1bs1b* (55.83%) and *m1bs1a* (53.29%) which was similar to our findings.

The* CagA*, *vacA s1a*, *m1a* and *m2* were the most commonly detected genotypes in the clinical food samples,^8^^,^^10^^,^^27^^,^^28^ those of animal sources^8^^,^^21^ and from human beings.^29^^-^^31^

Close association of *vacA* and *cagA* genotypes with production of interleukin-8 and cytotoxin, gastric epithelial cells adhesion, inflammatory effects, vacuolization and apoptosis in gastric epithelial cells has been observed previously.^32^^,^^33^ Since *H. pylori* isolates in our study harbored *vacA* and *cagA* genotypes, consumption of meat contaminated with virulent strains may provoke duodenal ulceration, gastric mucosal atrophy and gastric cancer.

In conclusion, Iranian meat samples harbored *H. pylori* strains with high prevalence of *vacA* and *cagA* genotypes. High prevalence of *H. pylori* in our samples suggests that contaminated meat samples maybe the sources of bacteria and can transmit to the human population. Diversity of *H. pylori *genotypes between various types of meat samples showed that there might be various sources of contamination. The most important finding of this study is that the meat samples of our research harbored virulent strains of *H. pylori*. A robust health protocol on hygiene of slaughterhouses and the butcheries can reduce the risk of transmission of *H. pylori* from meat and its products to human.
